# Treatment of orbital lymphatic malformation with oral sirolimus: a case report

**DOI:** 10.5935/0004-2749.20230072

**Published:** 2022-04-21

**Authors:** Carmen Alba-Linero, María García-Lorente, Rahul Rachwani-Anil, Guillermo Luque-Aranda, María-Isabel Pérez-Cabeza, Julia Escudero

**Affiliations:** 1 Ophthalmology Department, Hospital Regional of Malaga, Malaga, Spain; 2 School of Medicine, University of Malaga, Malaga, Spain.

**Keywords:** Lymphatic abnormalities/drug therapy, Orbit/pathology, Exophthalmos, Magnetic resonance imaging, Sirolimus/therapeutic use, Child, Anormalidades linfáticas/tratamento farmacológico, Órbita/patologia, Exoftalmia, Imagem por ressonância magnética, Sirolimo/uso terapêutico, Criança

## Abstract

It is estimated that lymphatic malformations in children account for 6% of all benign vascular malformations. New medical therapies have been developed for the management of lymphatic orbital disease. The purpose of this article was to describe a clinical case of orbital venolymphatic malformation in a 10-year-old boy, causing proptosis and palpebral edema. The lesion was initially treated with local sclerotherapy. However, the lesion relapsed, and was successfully treated with oral sirolimus. Prospective studies are warranted to determine the appropriate dose and extend the indications of sirolimus in these patients.

## Abstract (pt)

A incidência de malformações linfáticas em crianças é estimada em 6% de todas as malformações vasculares benignas. Têm sido desenvolvidos novos tratamento para doenças linfáticas orbitárias. Nosso objetivo é descrever um caso clínico de malformação venolinfática orbitária em um menino de 10 anos de idade, causando proptose e edema palpebral. A lesão foi tratada inicialmente com escleroterapia local. No entanto, a lesão teve recidiva e foi tratada com sucesso com sirolimo oral. Ainda são necessários estudos prospectivos para estabelecer a dose apropriada e a duração do tratamento com sirolimo nesses pacientes.

## INTRODUCTION

Lymphatic malformations, previously termed lymphangiomas, are benign malformations of the lymphatic system^(1)^.

There is an even distribution of lymphatic malformations among sexes and races. It is estimated that lymphatic malformations in children account for 6% of all benign vascular malformations; these conditions are usually diagnosed during the first years of life^(1)^.

Some conditions, such as infections, can trigger lymphatic vessel proliferation^(2)^. Occasionally, these lymphatic malformations grow proportionally to the growth of the body^(1)^.

Macrocystic lymphatic malformations contain cysts bigger than 2 cm. Microcystic malformations include cysts less than 2 cm or are characterized by soft tissue enlargement without cysts. Most orbital lymphatic malformations are mixed-type lesions, including both macrocysts and microcysts^(3)^. These are composed of thin-walled cystically dilated vascular channels lined by endothelial cells and filled with proteinaceous lymph fluid; they are also composed of lymphoid tissue. These low-flow vascular lesions are identified through ultrasound and magnetic resonance imaging (MRI)^(3)^.

The clinical manifestations of orbital lymphatic malformations are diverse and include signs (e.g., cellulitis, proptosis, and ptosis) and symptoms (ocular pain or diplopia)^(4)^.

The use of surgery for the treatment of lymphatic malformations remains controversial. Important structures often run within the septa of the lymphatic malformation. Hence, performing a whole resection in patients with extensive or infiltrative lymphatic malformations, while preserving non-pathological tissue, is particularly challenging^(4)^. Other treatment modalities, such as steroids or beta blockers and local sclerotherapy using various agents, have been tested. However, the recurrence rates observed following these treatments have emphasized the need for other therapeutic alternatives^(2)^.

Sirolimus (rapamycin) has been successfully used for the systemic treatment of lymphatic malformations. Therefore, it may effectively treat orbital lymphatic malformations^(5)^. The use of sirolimus in pediatric venolymphatic malformations is novel; this agent can be used alone or in combination with steroids^(6)^. Nevertheless, the dosage and timing of treatment are not established yet.

The aim of this article was to describe a clinical case of orbital venolymphatic malformation in a 10-year-old patient successfully treated with oral sirolimus.

## CASE REPORT

A 10-year-old boy attended our pediatric ophthalmology department with complaints about palpebral edema and proptosis of the right eye. His parents reported that the eyelid edema and proptosis had appeared 3 weeks earlier and that the symptoms were intermittent. The patient did not have other significant personal or family medical history. In addition, the patient did not have any past history of infection.

Visual acuity was 20/20 (Snellen chart) for both eyes. Intraocular pressure was 18 mmHg in both eyes. Examination through funduscopy did not yield any evidence of color changes or edema in the optic nerve.

The patient had evident right eye proptosis; measurement using a Hertel exophthalmometer revealed a difference of 3 mm between the eyes (i.e., 14 and 11 mm in the right and left eye, respectively).

Computed tomography revealed a high-density, intraconal, nodular lesion (27 mm) adjacent to the superior oblique muscle, causing proptosis and slight compression of the optic disc.

The ocular ultrasound showed a high-density collection located adjacent to the optic nerve with an increased vascular caliber suggestive of vascular malformation.

MRI confirmed an intraconal, post-septal, microcystic lesion with a venous vascular component in the right orbit (dimensions: 25 × 11 × 12 mm). These imaging analyses confirmed the presence of a diffuse, low-flow, intra-orbital, vascular lesion, probably corresponding to a multi-cystic venolymphatic malformation.

The patient was referred to another center for sclerotherapy using intralesional 2.2 cc bleomycin; this treatment resulted in a decrease in proptosis and eyelid edema.

**Figure 1:**
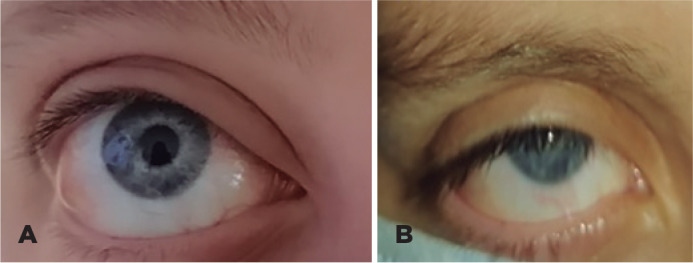
(A) External image of the right eye of the patient with lymphatic malformation revealing pre-septal inflammation, conjunctival chemosis, and proptosis. (B) External image of the same patient showing a decrease in orbital inflammation and proptosis 3 months after treatment with sirolimus.

One year after achieving anatomical stability, the patient returned to the clinic due to increased proptosis and discomfort in the right eye. He had developed severe proptosis with hypotropia of the right eye and accompanying eyelid edema (Figure 1A). Visual acuity, pupillary reflex, and extraocular movements were preserved, and intraocular pressure was normal.

**Figure 2:**
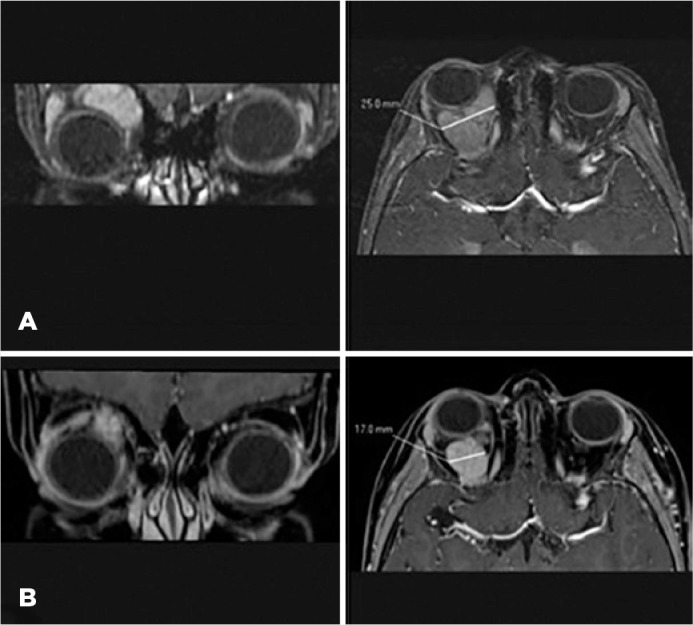
Cranial MRI, orbital section. Coronal (left) and axial (right) postcontrast SPIR T1WI. (A) Well defined and avid enhancing mass located in the right orbital space, consistent with orbital venous-lymphatic malformation. The lesion extends into the intraconal fat with shifting in associated structures resulting in proptosis (right) and infero-medial deviation of the right eye (left). There was no local infiltration observed. (B) Decrease in proptosis (left) and mass size from 25 mm (Figure 2A, right) to 17 mm (Figure 2B, right) after 3 months of treatment with sirolimus.

MRI, magnetic resonance imaging; SPIR, spectral presaturation with in­version recovery

Treatment with oral prednisone (initial dose: 30 mg) was initiated, and MRI was repeated. Imaging revealed an increase in the lesion size (dimensions: 25 × 31 × 33 mm) accompanied by intralesional bleeding (Figure 2A).

Subsequently, the patient was treated with oral sirolimus (1 mg, twice daily) (rapamycin, Rapamune^®^, Wyeth, USA). The drug was administered off-label, based on evidence of its use in this type of injury presented in the scientific literature^(5^,^7^,^8)^ Informed consent was provided by the patient’s parents prior to the treatment.

Three months later, the proptosis and hypotropia had subsided, and the patient was asymptomatic. In addition, the palpebral edema had resolved (Figure 1B), and the optic nerve remained normal.

The MRI results revealed the presence of a lesion with reduced size (dimensions: 17 × 15 × 15 mm) compared with that measured in the previous imaging analysis (Figure 2B). The patient did not develop any treatment-related side effects; at the last visit; the levels of sirolimus in his blood were 6.2 ng/ml.

## DISCUSSION

We describe a clinical case of venolymphatic orbital malformation in a 10-year-old patient successfully treated with oral sirolimus. Sirolimus has shown effectiveness in the systemic treatment of lymphatic malformations. Nevertheless, its use in the treatment of lymphatic malformations at the orbital level is limited. Furthermore, the appropriate dosage and duration of treatment are currently unknown.

Orbital lymphatic malformation is a rare disease (1%-3%) among orbital malformations^(2)^. The presence of clinical signs along with MRI can guide toward the diagnosis of such malformations. Definitive diagnosis can only be achieved by biopsy^(7)^; however, biopsies are technically difficult and invasive procedures.

Surgical treatment is challenging due to the location of these lesion. Hence, observation is often preferred in mild cases. Classically, sclerotherapy with various agents (e.g., bleomycin or ethanol) has been the therapeutic gold standard for more advanced lymphatic proliferations, and it is particularly useful in treating macrocystic malformations^(8)^.

However, sclerotherapy often does not achieve sustained effects over time or in cases of microcystic malformations (as in our patient). Moreover, it is associated with the development of serious adverse effects, such as severe local inflammation or compartment syndrome^(8)^.

Furthermore, treatment centers may not be able to offer different therapeutic options for this disease due to insufficient experience. In the present case, our patient was referred to another center for sclerotherapy with bleomycin.

A similar scenario occurs in treatment with sildenafil. Sildenafil is administered orally, does not have many contraindications, and is not linked to technical complications. Nevertheless, its efficacy has been demonstrated in macrocystic venolymphatic malformations, but not in microcystic malformations^(2)^.

Sirolimus is an inhibitor of the mammalian target of rapamycin (mTOR) that regulates several cellular processes. In a phase II clinical trial, Ricci et al. demonstrated its effectiveness in the treatment of generalized lymphatic anomaly^(9)^. Sirolimus is also an effective treatment option for isolated lymphatic malformations in other areas, such as the neck, mediastinum, or bone^(5^,^10)^.

As mentioned above, there is currently no consensus on the dosage and duration of treatment. In clinical trials, the target levels of sirolimus range 5-15 ng/ml^(2)^. The most frequent side effects of sirolimus include mild reversible leukopenia, hypertriglyceridemia, hypercholesterolemia, and hypertransaminemia^(5)^.

Some studies have reported positive results regarding the efficacy of sirolimus in the treatment of pediatric orbital venous-lymphatic malformations. Kim et al.^(6)^ reported a case of a venolymphatic malformation close to the optic nerve in a 2-week-old boy. The patient had been previously treated with sildenafil and sclerotherapy, and was successfully treated with oral sirolimus (0.8 mg/m twice daily) in combination with oral corticosteroids.

The successful combination of sirolimus with rivaroxaban for the treatment of a venolymphatic orbital malformation has also been described in a 20-year-old boy. In that case, the combination treatment reduced the size of the lesion and was not associated with thrombotic complications^(11)^.

Shoji et al.^(3)^ reported the most important case series of orbital lymphatic malformations in the literature. A total of 10 patients were treated (mean duration of treatment: 7 months; mean dose of sirolimus: 0.8 mg twice daily), achieving satisfactory results (i.e., reduction in the size of the lesions and good safety profile).

Lagrèze et al.^(12)^ described a case of retrobulbar lymphatic malformation treated with oral sirolimus (1 mg, twice daily) that resolved after 6 months. In another case, Lackner et al. extended the treatment to 10 months^(13)^.

In this case, the use of oral sirolimus resolved the patient’s symptoms 3 months after treatment. Specifically, after 3 months of treatment, the patient did not present hypotropia or proptosis, and a decrease in periorbital inflammation was observed. Through MRI, we also detected an objective reduction of the lymphatic malformation.

The dose used in this case (1 mg twice daily) was similar to that described by other investigators^(3^,^12^,^13)^ . This dosage did no lead to adverse effects; at the last visit, the levels of sirolimus in his blood were 6.2 ng/ml.

Therefore, sirolimus may be an effective and safe option for the treatment of pediatric lymphatic orbital malformations. Moreover, this therapeutic approach does not require a surgical technique the pediatric ophthalmologist. This avoids a learning curve process and the acquisition of required material. In addition, it is reasonable to state that medical treatment may be safer than invasive therapy in the pediatric population. Of note, the generalizability of these findings is limited by the involvement of a single patient and short follow-up period. Prospective studies involving pediatric patients with orbital lymphatic malformations are warranted to assess the long-term efficacy of sirolimus, determine the appropriate dosage, and extend its indications in these patients.
